# The Impact of Digital Contact Tracing Apps Overuse on Prevention of COVID-19: A Normative Activation Model Perspective

**DOI:** 10.3390/life12091371

**Published:** 2022-09-02

**Authors:** Junwei Cao, Dong Liu, Guihua Zhang, Meng Shang

**Affiliations:** 1 School of Business, Yangzhou University, Yangzhou 225127, China; 2 Department of Global Business, Yeungnam University, Gyeongsan 38541, Korea; 3 Department of Business, Yeungnam University, Gyeongsan 38541, Korea; 4 School of Flight, Anyang Institute of Technology, Anyang 455008, China

**Keywords:** digital contact tracing, normative activation model, COVID-19 prevention, prevention intention

## Abstract

During the COVID-19 pandemic, many countries have used digital contact tracing apps (DCTAs) to implement contact tracing. Although the use of DCTAs has contributed to the prevention and control of COVID-19, there are doubts in academia about their actual effectiveness. In this study, the role of DCTAs in the prevention of COVID-19 was analyzed in terms of both the responsibility and inconvenience to life in a large-scale DCTA overuse environment, based on the normative activation model. The findings suggest that the overuse of a DCTA activates people’s personal norms by triggering awareness of the consequences and ascription of responsibility, leading people to consistently cooperate with the government to prevent COVID-19. However, the inconvenience of living with DCTA overuse weakens the effect of the awareness of consequences and ascription of responsibility and the role of the ascription of responsibility in influencing personal norms. These effects may bear on people’s willingness to consistently cooperate with the government to prevent COVID-19. The results of this study confirm the effectiveness of DCTA in counteracting pandemics from a social responsibility perspective in a large-scale environment where DCTA is used, enriching the literature on DCTA research in the COVID-19 pandemic. The results of this study can also help governments develop and improve policies to prevent COVID-19, as well as improve the DCTAs’ operating patterns.

## 1. Introduction

Contact tracing of COVID-19 patients is a very important part of the global fight against COVID-19. Contact tracing, timely detection, and adequate isolation will play a significant role in slowing the spread of COVID-19 [1,2]. To implement contact tracing strategies, many digital contact tracing apps (DCTAs) were developed and widely used globally during the COVID-19 pandemic [3,4]. For example, Korea developed the “Self-isolation Safety Protection App”, Singapore the “TraceTogether”, Japan the “COCOA”, France the “StopCovi”, Germany the “Corona Warn”, and China the “Health Code” [5,6,7,8,9]. A DCTA will automatically record an individual’s travel history, and both the user and public health departments will be notified if the user enters a high-risk area or comes into contact with a suspected COVID-19 patient. The appearance of such apps is considered a public health intervention that could slow the spread of COVID-19 and save lives, as well as protect local health services [10,11].

Although DCTAs have been accorded high expectations, many studies have generally raised doubts about their effectiveness in the prevention of COVID-19 practices [10,12,13,14]. Many scholars believe that the premise for such apps to have an effect in helping prevent COVID-19 is the need for mass adoption and continuous use [15,16]; however, the DCTA adoption rate is too low to realize its full potential in most countries [17]. First, several studies have suggested that the inconvenience brought about by a DCTA to people’s lives affects its large-scale promotion, especially the privacy issue. A DCTA has issues with extensive personal information collection, multiple processing purposes, uncertain storage times, and vague privacy policies [9]. One survey claimed that many people in the UK refuse to use such apps because of privacy concerns [18]. A survey in Ireland noted that many people refused to use the app because they feared that tech companies or the government would use it to monitor users even after the COVID-19 pandemic was over [19]. Chinese users are concerned about the lack of transparency in the operation of DCTAs, the unclear scope of data storage, and the dependence on private companies to operate them [8]. South African users have also shown doubts about the app’s ability to protect privacy [20]. In addition to serious privacy issues, DCTAs have caused many other inconveniences, such as incorrect tracking and problems affecting the normal use of mobile phones [8,21]. Studies have shown that many of the close contacts located by a DCTA did not have any contact with COVID-19 patients, but they were also wrongly traced and isolated [8,22]. The operation of a DCTA has also been shown to affect the users’ normal use of their cell phones, such as reducing the phone’s running speed and affecting its battery life [21]. Second, several studies have suggested that the potential digital divide issue may also affect the large-scale promotion of a DCTA. A study shows that the digital divide during the COVID-19 pandemic often influences some people to use new technologies to prevent COVID-19 [23]. Age, education, income, health status, and regional differences can lead to a digital divide that directly affects people’s widespread use of the app [6,24]. A survey in Germany claimed that females and low-income households have lower rates of downloading the DCTA [6]. In a UK survey, it was found that the use of apps among those over 65 years old was low [25]. In addition, a study of the working population in Japan showed that the usage rate among small-company employees and vendors was low, while that among large-company employees and civil servants was high [7]. The reason this literature doubts the efficacy of DCTA is that most of the current literature consists of studies conducted in a free market environment where people who feel inconvenienced by the use of DCTA would simply stop using it without paying any price. However, research conducted in a government-led environment with large-scale mandatory DCTA use is missing.

In addition, it can be argued that the many inconveniences that a DCTA brings to people’s lives are the main issues that lead to doubts about its effectiveness. However, one study confirmed that the benefits of a DCTA could offset its negative effects [26]. The use of a DCTA has been shown to be closely related to individual as well as social interests [4]. This may be reflected in an individual’s sense of responsibility to family, friends, and the community (e.g., preventing transmission of the virus to others) [16,27]. Therefore, it is meaningful to explain the role of a DCTA in preventing COVID-19 in practice from the perspective of responsibility [11]. As more countries relax their control policies to prevent COVID-19, the trade-off between the benefits and negative effects of a DCTA will likely influence subsequent COVID-19 prevention behavior. The analysis of the role of a DCTA during the COVID-19 pandemic in terms of both responsibility and inconvenience helps resolve the doubts. Regrettably, such studies are currently lacking.

To address these issues, an environment needs to be found in which the social epidemic is relatively stable, while the use of a DCTA is still mandatory on a large scale. China happens to provide a very good environment for investigation. In terms of the people who use it, the DCTA in China is mandatory, and no one can refuse to use it [8]. In terms of the extent of use, the use of DCTAs in China is also widespread, with various DCTAs developed by the central government and local governments. Many Chinese people are already excessively using DCTAs, as they are required to register and show their tracking information when entering or leaving any place, taking public transportation, and traveling across cities. The overuse of technology often has a negative impact [28]. The overuse of DCTA technology in China does cause inconvenience to people’s lives, such as privacy concerns, data security, error tracking, etc. [8]. China is undoubtedly one of the most successful countries in the world in terms of its performance in preventing COVID-19 [29]. Therefore, this study proposes the following research questions:

RQ1: Does the overuse of a DCTA still inspire a sense of responsibility for COVID-19 prevention?

RQ2: How does the responsibility and inconvenience of the overuse of a DCTA affect people’s continued cooperation with the government to prevent COVID-19?

Therefore, this study builds its model based on the normative activation model (NAM) according to the actual research needs. People’s psychological states can be better measured by structured scales, and a number of NAM-based studies on COVID-19 have also used the questionnaire-based approach [30,31]. The model is then validated by surveying Chinese residents to ultimately address the proposed questions. The results of this study will not only help policy makers improve the operation of DCTA applications in the post-COVID-19 era of prevention and control but will also contribute to the improvement of national policies related to epidemic prevention and control.

## 2. Theoretical Background

The normative activation model (NAM) proposed by Schwartz [32] was used to explain altruistic behavior and was extended to explain various pro-social and pro-environmental behaviors, such as energy-saving behavior [33], green consumption behavior [34,35,36], environmental behavior [36,37], etc. The NAM has been widely used in environmental, psychological, and behavioral research and is among the most important theories for studying the individuals’ socially or environmentally responsible behavior [38]. During the COVID-19 pandemic, the NAM has been widely used in studies to analyze people’s infection prevention behavior. A study analyzed people’s willingness to get vaccinated before traveling from the perspective of the NAM, suggesting that mass media messages activated personal norms by positively influencing people’s awareness of the consequences and ascription of responsibility, prompting people to get vaccinated before traveling [30]. Another study analyzed Chinese people’s intentions to save masks in the post-COVID-19 era from the perspective of the NAM and confirmed that personal norms had a significantly positive impact on mask-saving behavior and that awareness of the consequences and ascription of responsibility indirectly influenced the intention to save masks through personal norms [31]. Therefore, it is appropriate to analyze the contribution of a DCTA to people’s willingness to consistently cooperate with the government in preventing COVID-19 from the perspective of the NAM.

**Figure 1:**
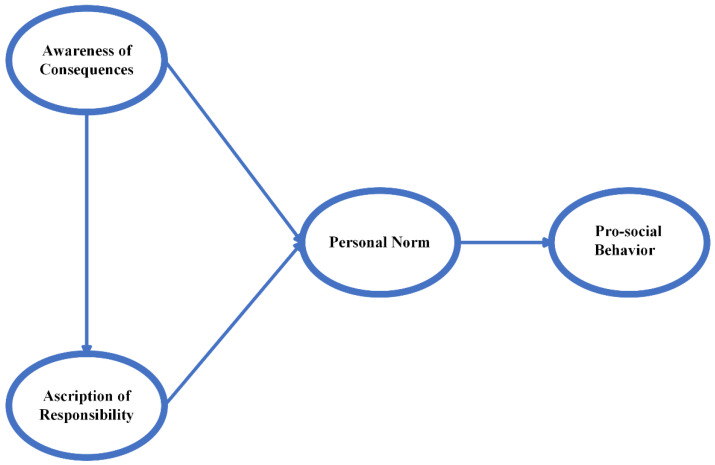
The original norm activation model.

The theory uses awareness of the consequences, attribution of responsibility, and personal norms to explain people’s pro-social behavior [32] (Figure 1). The NAM suggests that awareness of the consequences and ascription of responsibility can activate personal norms and thus trigger pro-social behavior. Pro-social behavior is an umbrella term covering a range of behaviors that have a positive impact on society, such as giving help, cooperating, and comforting [39]. Awareness of the consequences means that individuals are aware of the negative consequences of their actions [40]. Ascription of responsibility refers to the reflection of individuals who are responsible for the adverse consequences of their non-participation in pro-social activities [40]. Personal norms are defined as the moral obligations that a person needs to fulfill for a particular behavior. According to the theory, a person’s pro-social behavior or intentions are influenced by personal norms, and awareness of the consequences and ascription of responsibility can activate such norms [41]. People are more willing to engage in pro-social behavior when they perceive it as a moral obligation to perform or avoid a particular behavior [41,42].

## 3. Research Model and Hypothesis Development

**Figure 2:**
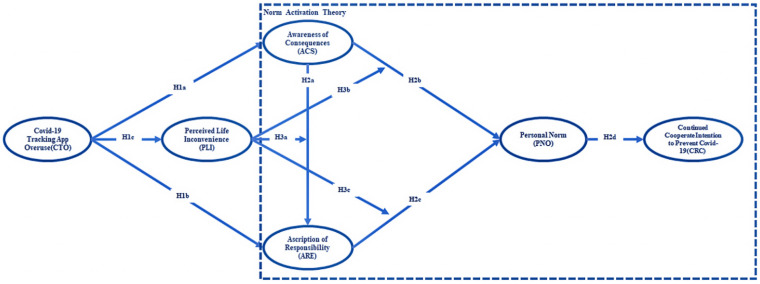
Research model.

Based on the NAM, this study develops a research model from the perspective of responsibility and inconvenience to people’s lives. It hypothesizes that the overuse of a DCTA can activate people’s personal norms by promoting awareness of the consequences and ascription of responsibility, and ultimately, a willingness to consistently cooperate with the government to prevent COVID-19. Meanwhile, the inconvenience caused by the overuse of a DCTA may affect people’s normative activation process. The resulting model is shown in Figure 2.

The monitoring of individuals will improve self-consequence management [43]. Therefore, the higher the perception of monitoring, the easier it is to perceive the consequences [44]. A DCTA’s tracking will make the users always feel monitored, while they will have to remind themselves to deal with possible consequences. For example, if individuals do not take precautions, they may be subject to prolonged isolation, more detailed epidemiological investigation, trajectory disclosure, and other mandatory measures if they become infected or come into close contact with a suspected COVID-19 patient [8]. A study in the UK confirmed that users were extremely concerned about privacy disclosure and stigmatization when using DCTAs during the COVID-19 pandemic [18]. Therefore, the overuse of a DCTA will prompt individuals to carefully consider the consequences and increase their awareness of them. Therefore, the following hypothesis is proposed:

**H1a.**  *The overuse ofDCTAs positively affects people’s awareness of theconsequences during theCOVID-19 pandemic*.

Monitoring and responsibility are closely related. A study suggests that parental monitoring activities can trigger students’ responsibility for learning [45]. It has also been noted that employee monitoring creates a sense of responsibility in managers [46]. In the prevention and control of the COVID-19 epidemic, the application of DCTAs can monitor the trajectory of each person’s action, while the government can be precisely responsible in the event of any trouble. A DCTA also serves an advocacy function that can remind people to take responsibility for their families and communities [16]. Thus, the overuse of a DCTA can leave users in constant fear of being held responsible if their actions have caused the spread of a virus. Therefore, the following hypothesis is proposed:

**H1b.**  *The overuse ofDCTAs positively affects people’s ascription of responsibilityduring the COVID-19 pandemic*.

The overuse of technology often has a negative impact [28]. A DCTA, a new technology arising from the COVID-19 pandemic, has been shown to create concerns about privacy, stigmatization, being mis-targeted, and data misuse [8,16,18]. Studies have pointed to concerns about the lack of transparency in the operations of DCTAs, the scope of data storage, the inability to change incorrect “red” codes (representing health risks), the over-reliance on the internet, and the reliance on private companies, such as Alipay and WeChat, to monitor their travel routes [8]. In addition, the overuse of a DCTA can lead to a need for people to present and register their travel tracks at any place, while individuals are often inconvenienced by the rapid changes in the epidemic and the inaccuracy of clients’ location displays for daily life. Therefore, the following hypothesis is proposed:

**H1c.**  *The overuse of DCTAs positively affects the inconvenience to daily life during the COVID-19 pandemic*.

The NAM proposes that consequence awareness has a significantly positive effect on the ascription of responsibility and that the two activate personal norms together [39]. Such a relationship has been prevalent during the COVID-19 pandemic. A study suggests that, in people’s willingness to be vaccinated, the awareness of consequences creates the ascription of responsibility, while the latter activates personal norms for vaccination [41]. Similarly, a study suggests that people’s awareness of the possible consequences allows them to actively take precautions while traveling in recognition of their potential responsibility in preventing COVID-19 [47]. One study analyzed people’s behavior during the waste sorting of masks during the COVID-19 pandemic and verified the roles of the awareness of consequences and ascription of responsibility in the activation of personal norms [48]. First, due to the overuse of DCTAs, people will feel that their travels are constantly being monitored and will have a strong awareness of the consequences. Second, the overuse of DCTAs allows for more precise accountability, while a sense of responsibility is attributed when people understand that they will be held accountable for the consequences they cause. Third, people’s concerns about the consequences and the ascription of responsibility together contribute to the creation of personal norms, which make people believe that cooperating with COVID-19 prevention is a moral imperative and pro-social behavior. Finally, due to the large-scale and continuous use of DCTAs, the mechanism of influence between awareness of the consequences, ascription of responsibility, and personal norms will persist and may facilitate people’s continuous intention to cooperate with the government in preventing COVID-19. Therefore, the following hypotheses are proposed:

**H2a.**  *The awareness of consequences that is caused by the overuse of DCTAs positively influences the ascription of responsibility*.

**H2b.**  *The awareness of consequences that is caused by the overuse of DCTAs positively influences the activation of personal norms*.

**H2c.**  *The ascription of responsibility that is caused by the overuse of DCTAs positively influences the activation of personal norms*.

**H2d.**  *Personal norms have a positive impact on people’s intention to consistently cooperate with the government to prevent COVID-19*.

The prospect theory proposes that individuals’ preferences and behavior under risk and uncertainty tend to follow an evaluation of their potential gains and losses and that people may be willing to take risks in exchange for benefits in the face of large, perceived benefits [49]. One study confirmed that during the COVID-19 pandemic, the perceived health and privacy risks jointly influenced the perceived benefits, while people would be willing to forego some life conveniences in exchange for health benefits [26,50]. Living with an inconvenience can have a negative impact on people’s behavioral intentions [51]. The overuse of DCTAs has caused many inconveniences in people’s lives, such as privacy issues and incorrect diagnoses [8]. However, such inconveniences can only weaken people’s willingness to continue to cooperate in the prevention of the disease and cannot be a direct deterrent. First, in an environment of government-led mass compulsory use, it is clear that people are more willing to endure the inconvenience of living with COVID-19 than to bear the consequences and responsibility of not cooperating in the prevention of COVID-19, although they are dissatisfied. Second, people use the new COVID-19 prevention technology because of their personal and community interests [4]. When people consider that the consequences of the spread of the epidemic may harm their personal or collective interests, they develop a sense of responsibility attribution, which makes them feel morally obliged to cooperate in the prevention of the epidemic, even if they are slightly dissatisfied. Overall, in the context of government-led mass-mandated use, the inconvenience of living with DCTAs is unlikely to directly affect people’s sense of responsibility, sense of consequence, and personal norms, but it can create negative emotions that may weaken the strength of the causality of the responsibility, sense of consequence, and personal norms variables, ultimately affecting people’s awareness of COVID-19 prevention. Therefore, the following hypotheses are proposed:

**H3a.**  *The inconvenience to life that is caused by the overuse of DCTAs weakens the contribution of consequence awareness to the ascription of responsibility*.

**H3b.**  *The inconvenience to life that is caused by the overuse of DCTAs weakens the role of consequence awareness in promoting personal norms*.

**H3c.**  *The inconvenience to life that is caused by the overuse of DCTAs weakens the ascription of responsibility in promoting personal norms*.

## 4. Method

### 4.1. Questionnaire Design and Survey

The scales for all the variables in the study were designed based on those that have been validated by existing studies. The scales used to measure the variables in the NAM were adapted from a related study conducted on the basis of the NAM (Sang, Yao, Zhang, Wang, Wang, and Liu [36], Kim, Woo, and Nam [38], and He and Zhan [34]). The scale for measuring overuse was adapted from Lee, Kim, Fava, Mischoulon, Park, Shim, Lee, Lee, and Jeon [28]; the scale for measuring the inconvenience to life was adapted from Lee, Kim, Fava, Mischoulon, Park, Shim, Lee, Lee, and Jeon [28]. After the initial questionnaire design was completed, we asked experts in the field to review and revise it and conducted a small-scale pre-test to improve it. Please refer to Appendix A Table A1 for specific measurement items.

Some of the other design parameters of the scale are as follows. (1) The scale uses a 5-point Likert scale. (2) The questionnaire questions are in English, while the survey was conducted in China; thus, we invited two linguists who were proficient in English to translate the questionnaire from English into Chinese to ensure that the Chinese presentation was error free and easy to understand. (3) We designed reverse questions in the questionnaire to detect invalid questionnaires. (4) Our questionnaire was designed as an anonymous survey, where participants were informed of the purpose of the study, only the necessary data were collected and kept strictly confidential, and respondents were given gifts to participate. (5) In accordance with the regulations of the Research Ethics Committee of Yeungnam University (https://irb.yu.ac.kr/02_gid/gid01.html, accessed on 20 June 2022), no specific ethical review was required for the questionnaire survey of this study.

**Figure 3:**
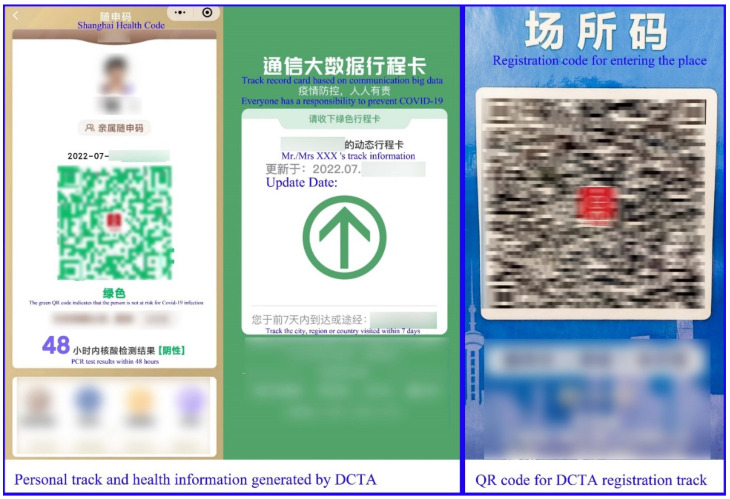
Tracking display/registration QR code generated by the DCTA in Shanghai.

We selected people living in Shanghai as the population for this study. First, Shanghai is a mega-city in China with a large population, a developed economy, and a rapid diffusion of new technologies and policies; the use of a DCTA to enhance health verification and entrance registration is an important initiative to strengthen COVID-19 prevention in Shanghai. Second, Shanghai had a massive COVID-19 outbreak in March, and after the pandemic was brought under control, the full deployment of “place code” and “health verification machine” devices was quickly made mandatory for citizens to use, while citizens had to scan the QR codes on these devices through their cell phones to complete health verification and tracking registration before entering places (see Figure 3) [52].

In this study, we randomly joined some instant messaging software chat groups in the Shanghai area and randomly conducted questionnaires among members of them. Participants who completed the survey would receive a CNY 10 shopping coupon. In total, 400 respondents living in Shanghai were randomly surveyed through various SNS platforms from 1 July 2022 to 10 July 2022. Finally, we received a total of 379 questionnaires and obtained 313 valid questionnaires (82.5%) by removing duplicate responses, biased reverse questions, and those with less than 2 min of answer time.

### 4.2. Structural Equation Model

We first used a descriptive analysis of the demographic characteristics of the sample. Second, we evaluated the indicators related to model quality. Finally, the proposed hypotheses were tested.

The covariance-based structural equation model (CB-SEM) and variance-based partial least squares structural equation modeling (VB-SEM) can both be used to analyze structural equation models. However, the following may be noted. (1) Partial least squares structural equation modeling (PLS-SEM) is more suitable than CB-SEM for measuring structural equation models with more than six latent variables [53]. (2) PLS-SEM is suitable for a wider range of data characteristics than CB-SEM, especially for handling non-normally distributed data [53]. (3) PLS-SEM is more suitable for small-sample measurements and exploratory studies [53].

This is an exploratory study with six latent variables in the research model and a small, effective sample size. Additionally, a multivariate normality analysis was performed on the data collected in this study using a web calculator to measure the distribution of the data (https://webpower.psychstat.org/, accessed on 13 July 2022). The results show Mardia’s multivariate skewness (β = 40.707, *p* < 0.001) and multivariate kurtosis (β = 473.530, *p* < 0.01) that suggest multivariate non-normality. In summary, PLS-SEM is more suitable for data analysis in this study [54,55].

## 5. Results

### 5.1. Demographics and Bias Test Results

**Figure 4:**
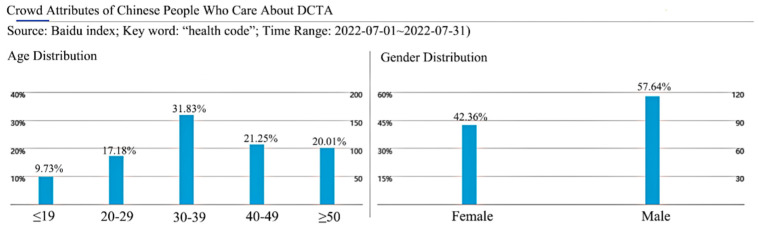
Crowd attributes of Chinese people who care about DCTA.

Among the 313 valid questionnaires collected from participants in this study, 125 (39.9%) were male and 188 (60.1%) female; the largest number of people were aged between 30 and 39 years (N = 165, 52.7%), followed by those aged between 40 and 49 years (N = 51, 16.3%). Of the participants, 172 (55%) had a bachelor’s degree, and 75 (24%) had master’s or doctoral degrees. The vast majority had a monthly income in the range of CNY 10,000–14,999 (N = 146, 46.6%), while 17.9% had a monthly income in the range of CNY 5000–9999 (N = 56). Referring to the data of people’s concern about DCTA in China from the Baidu Index (https://index.baidu.com, accessed on 19 August 2022) (Figure 4), this survey result has a certain degree of representativeness.

To detect the non-response bias, a paired *t*-test was performed on the demographic data of the first and last 25 participants in the survey [56]. The results of the test showed no significant differences. Therefore, non-response bias was not a serious problem.

Common method bias (CMB) is also a common problem in surveys, which we measured using two methods. First, this study measured the rate of extraction of single factors according to the method proposed by Podsakoff et al. [56], which was 24.154%, below the threshold of 40%. Second, this study was performed using the full-VIF method of measurement in PLS-SEM to detect CMB [57]. All VIF values were below the threshold of 3.3 [54]. The results of these two tests indicate that CMB was not a serious problem in this study.

### 5.2. Measurement Model Results

We measured the quality of the model by assessing the composite reliability (CR), average variance extracted (AVE), discriminant validity, and outer loading. As shown in Table 1, the CR and Cronbach’s alpha for all the variables exceeded 0.7, indicating that the internal consistency of the data in this study was satisfactory. The AVEs for the variables were all greater than 0.5, while the outer loading exceeded 0.7, indicating that the convergent validity of the data in this study was satisfactory [53].

We determined the discriminant validity using both Fornell and Larcker’s test and the heterotrait–monotrait ratio (HTMT) test. As shown in Table 2, the square root of each variable’s AVE is greater than the correlation with other variables [53]. The HTMT values were also all below 0.85. Therefore, the discriminant validity of this study is in accordance with the requirements [53].

### 5.3. Structural Model Results

Before measuring the structural model, we measured co-linearity (ensuring sufficient independence between variables), and the VIF for all the variables was below 3; thus, co-linearity was not a major issue in this study. After ensuring the reliability and validity of the model, we tested the hypotheses using the structural model. The path coefficients and significance test results from the structural model are shown in Table 3. Overuse of DCTAs had a positive and significant effect on awareness of the consequences, ascription of responsibility, and perceived life inconvenience, with H1a, H1b, and H1c being supported. Awareness of the consequences had a significantly positive effect on the ascription of responsibility and personal norms, thus supporting H2a and H2b. Ascription of responsibility had a significantly positive effect on personal norms, supporting H2c. Personal norms had a positive impact on the willingness to consistently cooperate with the government in COVID-19 prevention, supporting H2d. In addition, none of the control variables had a significant effect on the users’ intention to consistently cooperate with the government in COVID-19 prevention.

Finally, we evaluated the goodness of fit (GOF) of the model using the standardized root mean square residuals (SRMR). The SRMR value for the model is 0.068, which is less than the threshold value of 0.08. Thus, the fit of the model is satisfactory [58].

### 5.4. Moderating Effect Results

The perceived life inconvenience was used as a moderating variable; its moderating effect was measured through two steps in this study. First, we measured the significance of the moderating effect; second, we measured the strength of the moderating effect by calculating F^2^ as follows: (R^2^ interaction model − R^2^ main effects model)/(1 − R^2^ main effects model). If F^2^ is between 0.02 and 0.15, it indicates a small moderating effect; if it is between 0.15 and 0.35, it indicates a moderate moderating effect; and if it exceeds 0.35, it indicates a high moderating effect [59,60].

The moderating effects are shown in Table 4. The perceived life inconvenience significantly reduced the effect of awareness of the consequences on the ascription of responsibility (β = −0.158, *p* < 0.01), thus supporting H3a. Perceived life inconvenience also significantly reduced the effect of ascription of responsibility on personal norms (β = −0.158, *p* < 0.01), thus supporting H3c. However, perceived life inconvenience had no significant moderating effect on awareness of the consequences and personal norms, and H3b was rejected (β = −0.078, n.s.).

**Figure 5:**
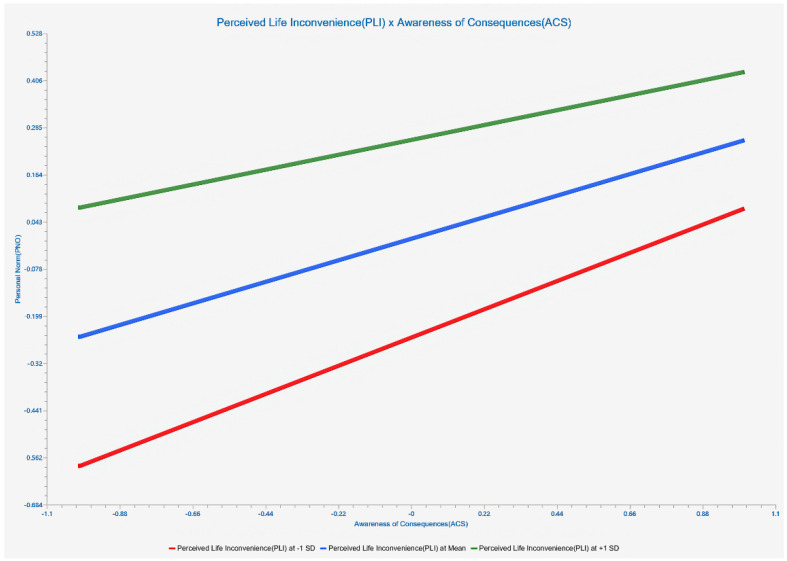
Simple slope analysis (PLI*ACS -ARE).

**Figure 6:**
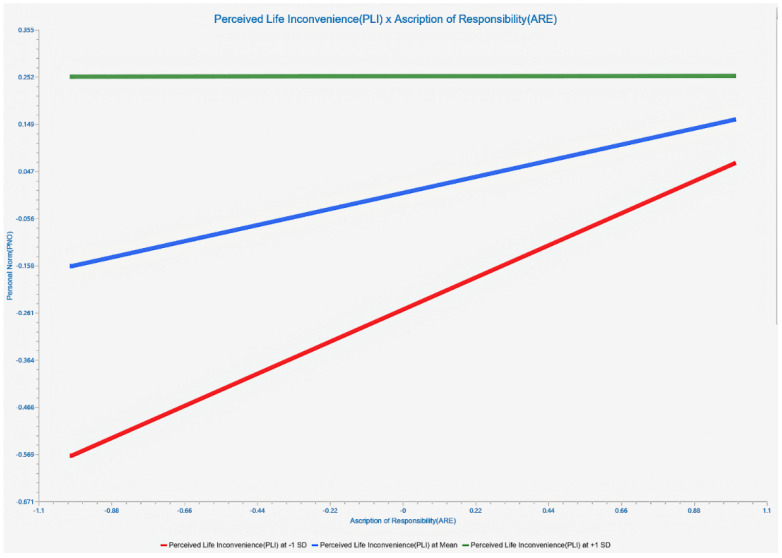
Simple slope analysis (PLI*ARE -NO).

Slope plots are provided as part of the moderating effect analysis to provide a more visual response to the enhancing/weakening effect of the moderating variable on a specific relationship. We performed slope analysis on the significant moderating relationships. The results are shown in Figure 5 and Figure 6. Perceived life inconvenience significantly reduced the predicted effect of awareness of the consequences on the ascription of responsibility, with a “medium” effect size (β = −0.158, *p* < 0.01, 0.02 < F^2^ = 0.023 < 0.15). Perceived life inconvenience significantly reduced the impact of ascription of responsibility on personal norms, with a “high” effect size (β = −0.158, *p* < 0.01, 0.35 < F^2^ = 0.072).

## 6. Discussion and Conclusions

### 6.1. Key Findings

The results of the study suggest that the overuse of DCTAs triggers awareness of the consequences and ascription of responsibility, that awareness of the consequences is an important antecedent of ascription of responsibility, and that the triggered awareness of the consequences and ascription of responsibility activate people’s personal norms. Guided by personal norms, people will continue to cooperate with the government to prevent COVID-19. Such results again validate the NAM theory in the context of the COVID-19 pandemic and are also in general agreement with the results of some studies on DCTA [8,10,11,18,21,30,31]. The tracking ability of a DCTA plays a role in monitoring people for the prevention of COVID-19. First, when a DCTA accurately tracks everyone’s travel trajectory and health status, people are worried about being held precisely accountable for the consequences of their bad behavior. Second, the lack of privacy protection in a DCTA may also cause people to worry that they may be stigmatized in the event that they are infected [18]. Finally, the deficiencies of a DCTA’s positioning accuracy can raise concerns [8,21]; people are urged to exercise caution to prevent being wrongly identified as a close contact and being investigated. The awareness of consequences and ascription of responsibility that people develop under DCTA monitoring will compel people to consider it a pro-social moral obligation to cooperate with the government to prevent COVID-19, and ultimately, to consistently cooperate with the government to prevent COVID-19.

The overuse of DCTAs has indeed also inconvenienced people in their lives, thus weakening their willingness to actively cooperate with the government in preventing COVID-19 (moderating effects of perceived life inconvenience). People use DCTAs in the spirit of personal and social interests [4]. However, it is human nature to “tend to benefit and avoid harm” [49]. The inconvenience of living with a DCTA can cause people to weigh the pros and cons of preventing COVID-19. Studies have demonstrated that people tolerate privacy risks in DCTA use when the privacy risks they pose are lower than the health risks [26,50]. It is reasonable to infer that when the inconvenience caused by a DCTA exceeds the level of responsibility required, people will choose to take responsibility rather than endure the inconvenience to life. In addition, when the inconvenience caused by a DCTA results in considerable losses, people may have the feeling that “DCTA has already caused me losses, so what is my obligation and responsibility to cooperate?” However, the reality is that due to the government’s strict precautions, the fear of being forcefully held accountable far outweighs the perceived inconvenience of living with a DCTA. Therefore, from the perspective of personal interest, even if people are dissatisfied, they are forced to develop a sense of responsibility and personal norms to cooperate with the government in preventing COVID-19 due to the awareness of the dire consequences. This explains the mechanism by which the inconvenience caused by the overuse of DCTAs plays a moderate-to-high intensity-weakening role in the relationships between awareness of the consequences and ascription of responsibility and between ascription of responsibility and personal norms; it does not directly negatively affect the relationship between ascription of responsibility and personal norms.

However, the debilitating effect of perceived life inconvenience from the overuse of DCTAs on consequence awareness and personal norms was not confirmed in this study. This inspires us to suggest that the inconvenient effects of a DCTA may need to be combined with precise accountability. The inconvenience caused by the overuse of a DCTA is magnified in the process of precise accountability, such as privacy concerns. When there is no accountability process, privacy is only restricted to a very few managers, and once the accountability process is involved, it can lead to stigmatization due to privacy breaches. Therefore, people will always carry this psychological pressure when using a DCTA. If the process of precise accountability is missing, a DCTA brings about only inconvenience to life, and people only need to measure the relationship between the inconvenience to mobility and prevention of COVID-19; they will naturally think that cooperating with the government to prevent COVID-19 is only a moral responsibility and will have no sense of responsibility for preventing COVID-19.

### 6.2. Theoretical Contributions

This study offers several theoretical contributions. First, this study evaluated the effect of DCTA overuse on promoting continuous cooperation with the government for COVID-19 prevention in a large-scale, mandatory-use environment and clarified the mechanism by which DCTA overuse promotes people’s cooperation with the government for disease prevention from a psychological perspective, thus enriching the literature on the effectiveness of DCTAs in disease prevention. Second, this study validated the issue of the feasibility of the NAM model in explaining people’s pro-social behavior in epidemics; it also verified that the overuse of digital health technology is an antecedent that triggers users to develop awareness of the consequences and ascription of responsibility, which expands the field of the use of the NAM and helps subsequent studies to apply it to investigate the impact of digital health technology on user psychology. Finally, this study verified the existence of a moderating effect of negative factors on the intrinsic mechanisms of the NAM model by analyzing the effects of perceived life inconvenience, which enriches the connotation of the NAM.

### 6.3. Practical Contributions

Our study also carries some practical implications for the prevention of COVID-19.

First, the government should not only develop functionally advanced DCTAs, but it should also have the ability to enforce their strict use nationwide. Meanwhile, the digital divide in the pandemic is worsening [23], making it difficult for many elderly and low-income people to use DCTAs because of accessibility issues. This requires the government to find ways to solve the tracking problem for this sector of the population, and the Chinese government’s practice in this regard is worth learning from and promoting. In many areas of China, people who do not have electronic devices and have difficulty using DCTAs only need to bring their ID cards when they travel, while public transportation drivers and staff in public places can use the “register and present for others” feature of a DCTA to help them overcome the digital divide caused by the adoption of digital technology.

Second, the tracking accuracy of a DCTA must be further optimized. This helps reduce the perceived inconvenience of life caused by DCTA. People worry about being wrongly isolated or pursued because of wrong tracking by a DCTA, which may be due to the layout of wireless base stations. When different areas are covered by the same base station, the tracking may be confused, resulting in location misclassification. Therefore, the developers of DCTAs should work with wireless network providers to optimize the location algorithm and base station distribution to reduce their chances of being mislocated.

Third, a special governmental supervision department should be established to implement confidential supervision of people’s tracking information through legislation and relevant technical measures and make timely adjustments to the errors that occur.

Finally, DCTA is only a precautionary measure that had to be taken to prevent COVID-19. The government should continuously adjust the level of DCTA use according to the changing situation of the COVID-19 pandemic. The inconvenience caused by the overuse of DCTAs to people’s daily lives and their negative emotions should be reduced.

### 6.4. Limitations and Future Directions

There are some shortcomings in this study. First, the survey in this study is limited to Shanghai, while the sample size is insufficient; thus, there may be some problems with representativeness, and future studies are encouraged to adopt more representative research methods, such as big data analysis of the epidemic. Second, the “perceived inconvenience” in this study is a broad concept, which can be further subdivided into travel inconvenience, privacy concerns, and so on, in subsequent studies. Finally, the number of elderly respondents in this study was small, while the elderly are considered to be a very important group in the prevention of COVID-19; it is expected that future studies on the use of DCTAs among the elderly can be conducted.

## Figures and Tables

**Table 1:** Reliability and validity of constructs.

Latent Variable	Item	Loading	Mean (SD)	Cronbach’s α	CR	AVE	R^2^
CTO	CTO1	0.841	3.143 (0.804)	0.819	0.866	0.618	-
	CTO2	0.821					
	CTO3	0.754					
	CTO4	0.731					
ACS	ACS1	0.851	3.028 (1.074)	0.839	0.903	0.757	0.111
	ACS2	0.915					
	ACS3	0.843					
ARE	ARE1	0.864	3.439 (0.653)	0.715	0.841	0.639	0.063
	ARE2	0.750					
	ARE3	0.780					
PLI	PLI1	0.923	3.149 (1.131)	0.898	0.93	0.769	0.020
	PLI2	0.758					
	PLI3	0.927					
	PLI4	0.889					
PNO	PNO1	0.883	3.544 (0.860)	0.818	0.888	0.727	0.125
	PNO2	0.894					
	PNO3	0.775					
CRC	CRC1	0.783	3.132 (0.714)	0.855	0.898	0.689	0.024
	CRC2	0.840					
	CRC3	0.781					
	CRC4	0.910					

Abbreviations: CTO—COVID-19 Tracking App Overuse; ACS—Awareness of Consequences; ARE—Ascription of Responsibility; PLI—Perceived Life Inconvenience; PNO—Personal Norm; CRC—Continue to Cooperate Intention to Prevent COVID-19.

**Table 2:** Discriminant validity.

Fornell–Larcker Criterion						
	ARE	ACS	PLI	PNO	CRC	CTO
ARE	0.799					
ACS	0.186	0.870				
PLI	0.114	−0.024	0.877			
PNO	0.247	0.294	0.253	0.852		
CRC	0.537	0.058	0.099	0.154	0.830	
CTO	0.222	0.333	0.447	0.266	0.174	0.786
**Heterotrait–Monotrait Ratio**						
	ARE	ACS	PLI	PNO	CRC	CTO
ARE						
ACS	0.236					
PLI	0.141	0.055				
PNO	0.304	0.332	0.294			
CRC	0.636	0.103	0.114	0.173		
CTO	0.224	0.338	0.443	0.301	0.151	

Abbreviations: CTO—COVID-19 Tracking App Overuse; ACS—Awareness of Consequences; ARE—Ascription of Responsibility; PLI—Perceived Life Inconvenience; PNO—Personal Norm; CRC—Continue to Cooperate Intention to Prevent COVID-19.

**Table 3:** Assessment of the structural model.

Hypothesis	β	STDEV	T-Statistic	*p*-Value	Result
H1a: CTO -> ACS	0.333	0.334	6.755	0.000	Support
H1b: CTO -> ARE	0.18	0.181	3.190	0.001	Support
H1c: CTO -> PLI	0.447	0.45	11.93	0.000	Support
H2a: ACS -> ARE	0.126	0.13	1.974	0.048	Support
H2b: ACS -> PNO	0.257	0.259	4.901	0.000	Support
H2c: ARE -> PNO	0.199	0.204	3.711	0.000	Support
H2d: PNO -> CRC	0.157	0.161	2.651	0.008	Support
Edu -> CRC	−0.016	−0.013	0.257	0.797	
Gender -> CRC	0.039	0.046	0.301	0.763	
Income -> CRC	0.035	0.032	0.505	0.613	
Age -> CRC	−0.039	−0.04	0.709	0.478	

Abbreviations: CTO—COVID-19 Tracking App Overuse; ACS—Awareness of Consequences; ARE—Ascription of Responsibility; PLI—Perceived Life Inconvenience; PNO—Personal Norm; CRC—Continue to Cooperate Intention to Prevent COVID-19.

**Table 4:** Moderation effects test.

Hypothesis	R^2^ Main Effects Model	R^2^ Interaction Model	F^2^	β	T-Statistic	*p*-Value	Result
H3a: PLI*ACS -> ARE	0.063	0.091	0.023	−0.158	2.796	0.005	Support
H3b: PLI*ACS -> PNO	0.125	0.215	-	−0.078	1.467	0.142	Reject
H3c: PLI*ARE -> PNO	0.125	0.215	0.072	−0.158	2.645	0.008	Support

Abbreviations: ACS—Awareness of Consequences; ARE—Ascription of Responsibility; PLI—Perceived Life Inconvenience; PNO—Personal Norm.

## Data Availability

The data presented in this study are available upon request from the corresponding author. The data are not publicly available for ethical reasons.

## Appendix A. Scale

**Table A1:** Scale.

Construct	No.	Item	References
Contact Tracing App Overuse (CTO)	CTO 1	I must use a DCTA to enter any place.	Lee, Kim, Fava, Mischoulon, Park, Shim, Lee, Lee, and Jeon [28]
	CTO 2	Even though using a DCTA to enter some sites caused queues, I had to use it.	
	CTO 3	I have to use a DCTA every time I travel.	
	CTO 4	Many places force me to use a DCTA.	
Awareness of Consequences (ACS)	ACS1	The overuse of DCTAs allowed me to understand the consequences of COVID-19 proliferation.	Sang, Yao, Zhang, Wang, Wang, and Liu [36] Kim, Woo, and Nam [38]
	ACS2	The overuse of DCTAs did not raise my awareness of preventing COVID-19 (Reverse).	
	ACS3	The overuse of DCTAs reminds me to avoid risky behaviors as much as possible.	
Ascription of Responsibility (ARE)	ARE1	The overuse of DCTAs has taught me that negative behaviors resulting in the spread of COVID-19 will be precisely pursued.	Kim, Woo, and Nam [38]
	ARE2	The overuse of DCTAs reminds me of my responsibility to cooperate in the prevention of COVID-19.	
	ARE3	The overuse of DCTAs makes me think that everyone must take responsibility for slowing the spread of COVID-19.	
Personal Norms (PNO)	PNO1	The overuse of DCTAs makes me feel obliged to cooperate in the prevention of COVID-19.	Kim, Woo, and Nam [38] Sang, Yao, Zhang, Wang, Wang, and Liu [36]
	PNO2	The overuse of DCTAs has forced me to cooperate in the prevention of COVID-19.	
	PNO3	The overuse of DCTAs makes me think it is morally responsible to cooperate in the prevention of COVID-19.	
Perceived Life Inconvenience (PLI)	PIC1	The overuse of DCTAs has caused inconvenience to my travel.	Seiders, Voss, Godfrey, and Grewal [51]
	PIC2	The overuse of DCTAs makes it inconvenient for me to get in and out of some places.	
	PIC3	The overuse of a DCTA adds inconveniences to my life, such as concerns about privacy leaks and being misplaced.	
	PIC4	The overuse of a DCTA forces me to spend a lot of time planning my life.	
Continuous Cooperation Against COVID-19 Intention (CAI)	CAI1	I intend to continue working with the relevant departments to prevent COIVD-19.	Kim, Woo, and Nam [38] He and Zhan [34]
	CAI2	I am willing to follow the guidance of the relevant department to continuously prevent COVID-19.	
	CAI3	Even if it costs me time and money, I am willing to keep working with the relevant departments to prevent COVID-19.	
	CAI4	I look forward to continuing to work with the relevant authorities to prevent COVID-19.	
